# Assistance for Folding of Disease-Causing Plasma Membrane Proteins

**DOI:** 10.3390/biom10050728

**Published:** 2020-05-07

**Authors:** Karina Juarez-Navarro, Victor M. Ayala-Garcia, Estela Ruiz-Baca, Ivan Meneses-Morales, Jose Luis Rios-Banuelos, Angelica Lopez-Rodriguez

**Affiliations:** Facultad de Ciencias Quimicas, Universidad Juarez del Estado de Durango, Durango C.P 34000, Mexico; karinajuarezn@hotmail.com (K.J.-N.); victor.ayala@ujed.mx (V.M.A.-G.); eruiz@ujed.mx (E.R.-B.); ivan.meneses@ujed.mx (I.M.-M.); qfbrios@gmail.com (J.L.R.-B.)

**Keywords:** proteostasis, quality control, misrouting, chaperones

## Abstract

An extensive catalog of plasma membrane (PM) protein mutations related to phenotypic diseases is associated with incorrect protein folding and/or localization. These impairments, in addition to dysfunction, frequently promote protein aggregation, which can be detrimental to cells. Here, we review PM protein processing, from protein synthesis in the endoplasmic reticulum to delivery to the PM, stressing the main repercussions of processing failures and their physiological consequences in pathologies, and we summarize the recent proposed therapeutic strategies to rescue misassembled proteins through different types of chaperones and/or small molecule drugs that safeguard protein quality control and regulate proteostasis.

## 1. Introduction

Currently, we understand the plasma membrane (PM) not as a simple lipid bilayer protecting the cells or surrounding the cytoplasm but as a collection of stably folded membrane proteins (MPs) in an asymmetric arrangement. In the PM, peptides interact with the lipid bilayer hydrocarbon core, the bilayer interface, and water in a minimum free energy state, forming complex and dynamic protein–lipid structures that participate directly as messengers or regulators of many signal transduction cascades. Regulation of these complex structures is essential for life and health [1,2,3]. MPs are difficult to study in vitro for many reasons, such as their flexibility, instability, and relatively hydrophobic surface. Since the first MP structure was published in 1985 [4], our knowledge has increased slowly but steadily. However, many aspects of the cell membrane are incompletely understood, including its lipid–protein organization and its stability to allow substances that meet strict criteria to transit unaided or through protein transporters, maintaining the intracellular/extracellular balance of substances according to physiological conditions.

Understanding how PM proteins are regulated from their synthesis to their final localization could provide further insight into the mechanisms of certain cellular events, such as folding, molecular sorting, and intracellular transport, that take place in lipidic membranes after protein synthesis. A wide range of genetic diseases is related to MP misfolding. Because the mutant proteins are either retained/accumulated intracellularly or are dysfunctional at the PM, signaling cascades mediating various physiological processes are mainly affected. In this review, we focus on PM proteins, analyzing their synthesis, folding, and trafficking in addition to the mechanism allowing their expression and stability at the membrane, and highlighting approaches by which natural and synthetic chaperones can be used to rescue misfolded phenotypes as strategic therapies to treat misfolding-related diseases.

## 2. Membrane Proteins

The external boundary of the cell is the plasma membrane (PM), and organelles are delimited by intracellular membranes. Lipids are essential components of all cell membranes. The composition of lipids and MPs may differ substantially depending on the function, organelle type, cellular location, or tissue level with which they are associated [1,2,3]. In many cells, phospholipids (glycerophospholipids and sphingolipids) are the cellular building blocks, while non-phospholipids are important regulators of lipid organization [1]. Cholesterol is the most abundant non-phospholipid in mammalian biomembranes. In human brain cells, cholesterol is a major constituent; it is critical for brain development. Cholesterol depletion leads to central nervous system pathologies such as Huntington’s [5] and Alzheimer’s [6], among other diseases [7,8]. Lipid content of PM influences the ion channel electrostatic environment, leading to an indirect modulation of ionic current and charge movement by modifying the transmembrane. Different studies have experimentally demonstrated that voltage-gated potassium (Kv) channels are sensitive to cholesterol [7,8,9,10,11] and phospholipid content [12,13,14,15]. 

Indeed, lipid distribution is highly regulated and not random across different membranes as phosphoinositides (PIs) show a clear demarcation in the cell. The eight members of the mammalian PI family (PI, PI3P, PI4P, PI5P, PI(4,5)P2, PI(3,4)P2, PI(3,5)P2, and PI(3,4,5)P3) play critical roles in modulating biological processes, such as gene expression, signaling, and membrane and cytoskeletal responses, and in membrane trafficking, among others. Thus, whereas some members, such as PI3P and PI(3,5)P2, along with their effectors and specific phosphatases are mainly present at the early and late endosome, respectively [16], PI(4,5)P2 is essential for endocytosis, exocytosis, and the regulation, adhesion, and assembly of membrane proteins [17,18]. Because of the specific distribution of PI family members in the cell, it is reasonable to fulfill important roles for MP location and function. Recently, multiple roles of lipids in ion channels and transporter regulation have been demonstrated, highlighting the importance of lipid–protein interactions for the cell physiology [19,20].

The abundance of MPs in the cell is low compared to the total protein population; the proportion of putative MPs predicted from sequenced genomes is between 20% and 35% [21,22]. PM proteins carry out diverse functions such as transporting nutrients to cells, regulating the exchange of bioactive molecules and receiving chemical signals from the extracellular space, activating signaling pathways in response to different stimuli, allowing the translation of chemical signals into intracellular activity, enhancing intercellular interactions, and sometimes promoting cell anchoring in a particular location [23,24,25].

MPs are grouped into two broad categories based on the nature of their interactions: (1) Integral MPs, also called intrinsic proteins, are integrated into the membrane; many of them can span the entire lipid and include more than one linked transmembrane domain fully embedded in lipid bilayers (also called transmembrane proteins). Examples of integral MPs include aquaporins, ion channels, transporters, and pumps. (2) Peripheral MPs, or extrinsic proteins, are entirely outside the membrane, indirectly bound to it by weak molecular interactions (e.g., ionic, hydrogen, and/or Van der Waals bonds), with integral MPs or the polar head groups on lipids. These MPs include phospholipase C, alpha/beta hydrolase fold, annexins, and synapsin I [26,27,28].

The mechanisms governing the selection and localization of PM proteins are strongly controlled; MPs have additional sequences (e.g., stop–transfer sequence membrane-spanning regions, glycosylphosphatidylinositol (GPI) anchors) that allow their integration into endoplasmic reticulum (ER) membranes. As reticular membranes move to the Golgi apparatus and finally to the PM, transmembrane proteins, such as ion channels and transporters, remain integrated with the internal membranes and then associate with the external membrane or stay partially embedded in one leaflet of the bilayer [29,30,31,32]. Caveolins (Cavs) are a good example of integral proteins partially embedded in one leaflet of the bilayer. The amino and carboxy terminal domains of Cavs flank a central hydrophobic region; hence, the formation of a hairpin in the lipidic bilayer is suggested by the aminoacidic sequence of this protein. Cavs are essential proteins for the formation of PM invaginations called Caveolae (“little caves”). The co-translational ER membrane insertion of Cavs depends on the signal recognition sequence [33,34]. Cavs are exported in a vesicle-dependent manner from the ER to Golgi. After post-translational modifications, caveolae transport oligomeric Cavs embedded into cholesterol-rich membranes. At the cell surface, Cavs interact with protein adaptors (Cavins) to assist membrane curvature. The lipidome of caveolae depends on the cell type and physiological condition. Lipid–protein interaction in caveolae may influence protein conformation, protein interactions, or ligand affinity, affecting cascaded signal transduction and cell physiology [35].

### Protein Biosynthesis

When mRNA reaches the cytosol, two ribosomal subunits associate with the initiation codon through eukaryotic initiation factors (eIFs) to integrate the translation initiation complex [36]. After the signal peptide denoting an MP is detected by the protein/RNA complex—called signal recognition particle (SRP)—the new protein will be inserted into the ER membrane, mediated in a co-translational or post-translational manner [37]. In general, all MPs are assembled in the ER, achieving their tertiary and quaternary structures.

In the co-translational pathway, many MPs and secretory proteins are formerly expressed as a pre-protein with an N-terminal topogenic sequence, which is a crucial signal peptide for protein localization and for protein insertion and orientation in cellular membranes. Commonly, 15 to 30 amino acids conform to the signal peptide; however, length can be up to 50 residues. This peptide sequence is typically cleaved off co-translationally [38]. Table 1 displays several signal sequences reported for proteins located in the ER and PM.

In general, even when sequence conservation among signal peptides is low, they have common secondary structural features, including a hydrophilic region at the N-terminal—which is frequently positively charged, a central hydrophobic domain, and a site for signal peptidase cleavage located in the C-terminal region; signal peptides can also display extended N-regions or hydrophobic regions [38,53].

After the signal peptide denoting an MP is detected by the SRP, it interacts with the SRP receptor in a GTP-dependent manner and undergoes several structural modifications. Then the SRP–ribosome complex docks to the translocon—a channel composed of proteins crossing the lipid bilayer to the ER lumen [10,17,18,19,20,21]—and translocation is resumed. the translocon is closed and the ribosome dissociates. The nascent protein is unloaded into the translocation channel (or translocon), GTP hydrolysis occurs, and the SRP receptor is free for another cycle [41]. Along with peptide translocation, signal peptide cleavage is mediated by the signal peptidase complex. Cleavage frequently occurs in a co-translational way [42,43,44], but it can also happen at some point between the early co-translational and late post-translational stages [45]. When translocation finishes, the translocon is closed and the ribosome dissociates.

When the ER protein in the lumen is translocated across the membrane, the protein is shifted laterally for anchoring within the phospholipid bilayer, allowing the protein to be integrated or assembled with other proteins into the ER membrane [54,55]. During translocation, enzymes (e.g., signal peptidases and oligosaccharyltransferase) can associate with the protein to cleave the signal peptide or N-glycosylate the translocating nascent chain [56,57].

In a conventional protein trafficking pathway after ER processing, vesicles favor the anterograde protein traffic toward the Golgi apparatus to further fuse transport vesicles with the target membrane, allowing protein insertion into the PM. Vesicular trafficking also occurs in an anterograde way to support PM protein quality control and cell integrity. A growing number of proteins have been associated with an unconventional protein trafficking pathway where proteins are delivered to the surface of the PM in an ER- or Golgi-independent manner. Exceptionally, most of the proteins following the unconventional trafficking pathway lack the classical N-terminal signal peptide, although if the signal peptide is recognized, proteins bypass the Golgi to reach the PM or be excreted [58].

Particularly, the C-tail anchored MPs—called tail-anchored (TA) proteins—constitute 3%–5% of the eukaryotic membrane proteome [37,59]; they lack the classical N-terminal signal for membrane insertion because they constitute a single stretch of hydrophobic amino acids where the membrane-interacting region is near the COOH terminus. After translation ends, a hydrophobic region is recognized and captured by specific cytosolic chaperones forming a pre-targeting complex that drives the opening of an ER membrane receptor, allowing TA protein insertion into the ER by a GTPase-dependent step [37,60,61]. TA proteins are found essentially in the intracellular membranes, carrying out various enzymatic and regulatory functions in the cellular metabolism, including apoptosis and protein quality control processes, besides protein localization and membrane traffic [62].

## 3. Membrane Protein Folding

In addition to the folding guided through the amino acid sequence, cytosolic regions may interact with intracellular proteins or chaperones for proper folding. Molecular chaperones are proteins that interact with a nascent protein to assist the stabilization of the native conformation, allowing the protein to remain in the intermediate states for longer during the folding process, but chaperones are frequently absent in the final functional structure [63]. The major ER-resident chaperones are proteins of the heat shock protein (Hsp) family [64], lectin chaperones, calnexin, and calreticulin [65].

Several Hsps are usually located in the ER; however, different types and levels of chaperone genes can be expressed under stress conditions or indifferent cellular stages [65,66,67,68,69,70]. 

In addition, chaperones assist the translocation machinery and play a role in the retrograde transport of aberrant proteins destined for proteasomal degradation [70,71], regulating the unfolded protein response (UPR) [72]. 

Along with an abundance of cytosolic and ER-specific molecular chaperones, the ER contains several classes of folding enzymes. *Prolyl cis-trans* isomerases, which via *cis-trans* isomerization of proline residues constrain the flexibility of the peptide backbone favoring the formation of disulfide bonds, catalyze co-translational and post-translational modifications important for protein folding [73,74,75]. Different kinds of chaperones are dissociated or associated with proteins throughout the protein maturation process [76,77]. 

Although an entire machinery of molecules participates in the protein folding process, misfolding sometimes happens despite the cell efforts to prevent it. Mutations and protein translation errors are frequently associated with protein misfolding. Nevertheless, different factors (e.g., age-related errors, exposure to environmental stress conditions, or lack of chaperone availability) can also induce aberrant folding [78]. To avoid misfolded protein aggregates, cells have developed sophisticated quality control mechanisms preventing dysfunctional proteins from reaching their destination. Control quality mechanisms are mediated by chaperone-dependent disaggregation and refolding systems and/or systems regulated through selective proteolysis. However, when a system fails, misfolded toxic aggregates lead to severe human diseases, such as neurodegenerative diseases (e.g., Alzheimer’s, Parkinson’s, Creutzfeldt–Jakob, and Huntington’s), diabetes, and cancer, among others [79,80,81].

The structural integrity of proteins must be constantly monitored by quality control mechanisms throughout their life—from translation in the ribosome to their arrival at the functional location in the cell. Once the proteins reach their final destination, a subsequent monitoring system ensures protein integrity; if at some point in their life cycle, proteins are recognized as terminally misfolded, they will be eliminated.

Proteins are synthesized on cytosolic ribosomes, but the ER is the main entry gate for secretory proteins expressed in intracellular organelles (including the ER), the PM, and cellular exterior. Hence, the ER features the first-line cellular quality control system (QCS) as it must ensure proper folding and assembly for proteins [63,82,83].

### 3.1. Quality Control Systems for Membrane Protein Folding

Cells have different QCSs to remove unfolded proteins. The UPR is a fundamental signaling pathway to keep cell homeostasis; through this pathway, unfolded proteins are exported from the ER and degraded in lysosomes, therefore increasing the ER folding capacity. When misfolded MPs are retained in the ER, the latter becomes stressed, and the UPR pathway is activated. The UPR comprises multiple strategies acting in parallel and/or in series to restore normal ER functioning [84]. 

In response to ER stress, major branches of the UPR are activated with the following aims: (1) increase the biosynthetic capacity—upregulate the expression of ER-resident chaperones—to prevent protein aggregation and facilitate correct protein folding; (2) control transcription, regulate mRNA abundance by stimulating or inhibiting transcription or by enhancing or compromising mRNA stability; (3) decrease the biosynthetic burden (attenuate translation) to reduce the transit of proteins through the ER, while the synthesis of membrane lipids increases the ER volume; (4) translocate misfolded proteins out of the ER; (5) remove misfolded proteins within the ER (by lysosomal/proteasomal degradation or ER autophagy) [85,86].

These branches include at least three mechanistically different components of the UPR: the RNA-dependent protein kinase-like ER kinase (PERK), activating transcription factor 6 (ATF6), and inositol-requiring ER-to-nucleus signal kinase 1 (IRE1). Coordinated actions of these proteins modulate gene expression, affecting the synthetic and secretory pathways, cell fate, and the metabolism of proteins, amino acids and lipids by activating specific transcription factors (e.g., ATF4, ATF6N, and X- box-binding protein 1 [XBP1], respectively) to lower ER stress [87]. A wide range of dysfunctions would otherwise be lethal if not for this intervention. When it becomes clear that a misfolded protein cannot be properly refolded, cellular stress persists, and the UPR negatively impacts the health of the cell and induces apoptosis [88,89,90]. As part of the UPR response, the transmembrane protein kinase PERK inhibits the translation of new proteins. After sensing ER stress, oligomerization of the luminal domain (N-terminal) of PERK facilitates autophosphorylation. After PERK is processed, it phosphorylates the α subunit of eukaryotic initiation factor 2 (eIF2α), which induces a transient attenuation of protein translation along with the activation of stress-responsive transcription factors to stimulate the expression of chaperones, oxidative response genes and autophagy/apoptosis genes, among other UPR-related proteins [53,54,55,91,92].

IRE-1 represents the most conserved signaling pathway. Through this pathway, chaperone expression is increased to modulate the ER-associated degradation (ERAD) pathway, responsible for a common process that clears the ER from potentially harmful species. ERAD machinery drives the retrotranslocation of misfolded proteins to the cytosol, where ubiquitin/proteasome proteolysis occurs [78,89]. ATF6 and IRE-1 interaction regulate the quantitative and qualitative expression of XBP1 to compensate for unfolded protein accumulation. After the UPR response is activated, ATF6 is transported to Golgi, where it is further enzymatically cleaved. The released fragment acts as a transcription factor that regulates the expression of proteins such as IRE1 and BiP chaperone [93,94]. 

Unfolded proteins stuck in the ER are eventually degraded; therefore, they must be retranslocated into the cytosol. Proteins require a signal, like ubiquitination, to be recognized for degradation, thus ensuring their delivery to the proteasome. However, a peptide signal for degradation is unclear. Experimental evidence shows that some molecular chaperones or protein disulfide isomerase homologs can associate with degradation substrates to prevent aggregation and escort selected polypeptides from the ER to the proteasome or lysosomes [95]. After retrotranslocation, several ubiquitin-binding proteins guide degradation substrates to the proteasome [96]. Even when glycosylation and protein degradation are evidently associated, it is less clear how non-glycosylated proteins are recognized for degradation. As the efficiency of binding through the calnexin/calreticulin cycle is dependent upon the oligosaccharide structure, proteins that are not properly glycosylated may activate a degradation pathway known as enhancing α-mannosidase-like protein (EDEM) [97]. Since the membrane protein degradation mechanism is not yet understood, it may start at the protein soluble parts after dislocation from the retrotranslocon or through a direct protein excision from the lipidic membrane. Analysis of MP degradation fate demonstrated that undegraded molecules accumulate in the cytoplasm when proteasome function is compromised, suggesting that transmembrane segments might be solubilized from the ER membrane before proteasome-mediated degradation [98].

Along with protein degradation mediated by proteasomes or lysosomes, ER autophagy (ER-phagy) can occur as an alternative to de-stress ER, removing aberrant portions of the ER containing abnormal proteins [99,100]. ER stress-mediated autophagy is a cellular catabolic process in which misfolded proteins, protein aggregates, and damaged ER regions are transported to the lysosome for degradation. Lysosomal degradation is induced by a set of hydrolases working in an acidic environment; afterwards, building blocks are recycled by the cell. The mechanisms by which MPs are tagged for lysosomal degradation are known when the translocation of cytosolic proteins is associated with a specific degradation signal (the KFERQ sequence motif) [101].

Evidently, the UPR response affects not only ER-related proteins but also genes associated with different cellular processes, including metabolism and inflammation; thus, gene regulation cannot always be explained through a canonical mechanism that only considers ER factors directly affecting protein processing [102,103]. UPR transcriptional output can be supported by parallel non-canonical stress-sensing mechanisms that may modulate the canonical mechanism. The translation of proteins that are not related to ER function may be implicated, as well as the self-association of proteins creating stress-specific scaffolds integrated by a multimeric protein complex. ER stress can also induce transcriptional cascades, expanding the expression of UPR gene regulatory networks and regulatory complexes not expressed constitutively. For example, the association of PERK and IRE-1 may create stress-specific scaffolds, inducing splicing mechanisms on XBP1 protein expression and affecting the transcriptional regulation [104]. IRE-1 can also recruit the TNF receptor-associated factor 2 (TRAF2), inducing activation kinases (e.g., IKK, JNK, ASK1, p38 MAPK, and ERK) that are important to determine the cell´s fate (survival/apoptosis) [105]. Another emerging point of regulation is related to microRNAs, the short (∼22 nt), single-stranded RNAs binding to complementary mRNAs to inhibit protein translation [106]. In general, the expansion of UPR signals impact cell fate.

To prevent issues related to the quality control of protein folding, the cell developed QCSs beyond ER checkpoints; for instance, in the Golgi apparatus, Rer1 and ERp44 proteins recognize immature proteins and facilitate their retrograde trafficking to the ER [107,108]. After passing the Golgi QCS, MPs embedded into transport vesicles can continue towards their functional locations. PMs also seem to have their own QCSs, including endocytic adaptors and ubiquitination systems, to monitor the MP structural integrity [109]. 

**Figure 1:**
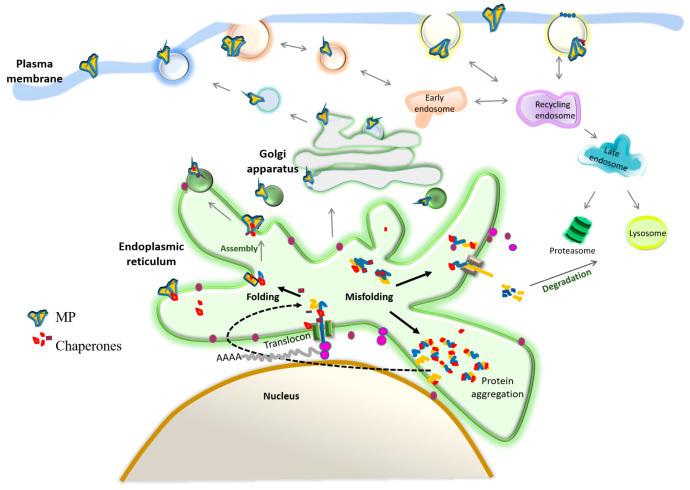
Trafficking of membrane proteins. As soon as the new membrane protein (MP) starts translocating to the ER, it will associate with chaperones and other proteins, assisting in getting a structural conformation into the lipid membranes. Well-folded proteins will continue trafficking through the endomembrane system to and from the PM, while misfolded proteins are re-directed to ER protein folding or degradation pathways, reducing their secretion to the extracellular space where they could further misfold or aggregate into proteotoxic conformations.

Therefore, intracellular trafficking is highly regulated, even when proteins may have particular motifs for exportation. If a QCS detects structural frustration indicating misfolding [110], proteins will be stuck in traffic. Misfolded proteins can undergo a refolding process, although proteins unable to fold may induce ER or mitochondrial stress, activating the degradation pathway to release the stress. After passing the QCS at the ER and Golgi, protein composition at the PM represents a complicated balance of membrane delivery, endocytosis, and recycling mechanisms [82] (Figure 1).

Long- and short-distance communication can take multiple vesicular forms, generally created through a membrane budding and pinching off mechanism. For MPs, vesicular transport is a milestone to safeguard proper folding and integrity while trafficking to the final destination. After ER processing, proteins are transported by coated vesicles (60–90 nm in diameter). The coat is a protein complex integrated by four subunits: Sec23/24-Sar1 selects cargo while Sec13/31 deforms the membrane to induce the budding. Some of the proteins incorporated into the vesicles can act as receptors for soluble proteins, thus favoring their packaging. After the vesicle buds from ER, translocation to the Golgi apparatus occurs, allowing post-translational modifications [111,112,113].

All cells contain a subset of membranous vesicular/tubular carriers formed by a direct budding of membranes. Vesicles are responsible for several cellular processes involving intracellular trafficking, including endocytosis and exocytosis. Primary endocytic vesicles can fuse with early endosomes to continue toward the protein maturation process—via a constitutive recycling pathway—or to prepare them for transportation to lysosomes [114]. Vesicles can be classified into three groups based on size and biogenesis: Apoptotic bodies released as an apoptotic response (800–5000 nm in diameter), ectosomes released directly from the PM (100–1000 nm in diameter), and exosomes originated from the inward budding of endosomes into multivesicular bodies (late endosomes; 30–100 nm in diameter) [115]. Despite the vesicular origin and size, there is still no reliable way to distinguish between ectosomes and exosomes, and the function may be quite analog. Particularly, some of the exosomes carry cargo molecules directly into the lysosomal compartment for degradation. Exosomes also form intraluminal vesicles to transport molecules (e.g., proteins, RNA [116,117] or Hsp chaperones [118]), which along with the cargo of extracellular vesicles may affect organelle function or modulate recipient cell function, thus contributing to the molecular intercellular transmission [119]. The recognition and packaging of cargo proteins result from multiple cooperative interactions between accessory proteins and lipids. Distinct ER-to-Golgi forward-trafficking signals have been identified on cargo or cargo receptor proteins. In potassium channels (Kir 1.1 and Kir2.1), the export signaling motif is not required for channel folding, assembly, or gating, but it is crucial for ER export [120].

### 3.2. Membrane Protein Modifications

After the new proteins are correctly folded and assembled in the ER, they travel towards the Golgi apparatus into transport vesicles, where they usually undergo different types of post-translational processing; however, protein modifications occurring co-translationally in the ER or post-transductionally in the Golgi apparatus also play an integral and crucial role in protein trafficking and function [112].

Some protein modifications, such as glycosylation, can reduce protein dynamics by increasing stability and favoring protein trafficking. N-glycosylation is the most common process to add sugar moieties to proteins. Glycosylation often starts while MP is translocated into the ER, and a preassembled polymannose oligosaccharide is transferred to the luminal N-aminoacidic residue of the classic motif including asparagine-X-serine/threonine(N-X-S/T) as a consensus sequence. Once in the Golgi, some enzymatic reactions can add sugar moieties or just modify the preexisting glycan tree complexity [121,122,123]. 

Phosphorylation is the enzymatic transference of phosphate from the ATP molecule on the side chains of serine, threonine, or tyrosine residues. Phosphorylation can occur co- or post-translationally in a reversible process affecting a wide variety of processes, including protein trafficking, clustering, conformation, and protein–protein interactions [124]. There are a few well-studied phosphorylation-induced trafficking examples. For instance, serotonin transporter (SERT) trafficking can be regulated when serine and threonine phosphorylation favors the recruitment of membrane skeleton adaptor protein Hic-5, inducing the actin-dependent endocytosis [125,126,127]. Exceptionally, phosphorylation at tyrosine residue in Kv1.3 triggers opposite effects depending on the protein life-stage, inducing either protein surface targeting or endocytosis of the channel [128].

The formation of reactive oxygen and nitrogen species (ROS/RNS) normally induces the post-translational oxidative modification of proteins, usually affecting cysteine residues due to their highly reactive thiol group. ROS/RNS can affect the thiol group of cysteine residues. In the mammalian proteome, two amino acids contain sulfur residues: cysteine and methionine. Particularly, the thiol group of cysteine enables multiple oxidation states allowing redox modifications that contribute to the signaling cascade specificity [129]. Many types of cysteine oxidative modifications can be reversed depending on the physiological condition of the cell [130]; protein modification through cysteine residues senses and transduces signaling cascades and regulates biological outcomes. Post-translational modifications like phosphorylation, glycosylation, and ubiquitination can combine with redox regulation to control cellular physiology. Even though many redox mechanisms affecting PM protein trafficking are still unknown, these mechanisms are interesting and promising, especially to understand the progression of many pathologies such as cancer and cardiovascular diseases [131].

At least six types of lipids, including fatty acids, sterols, isoprenoids, GPI anchors, and lipid-derived electrophiles, can attach to the cysteine, serine, or lysine residues of proteins. The covalent but reversible attachment of fatty acid with cysteines via a thioester linkage is called S-acylation, whereas the covalent interaction between lipids and proteins is called protein lipidation. This process occurs co- or post-translationally, and its deregulation has been linked to different diseases, including metabolic diseases, neurological disorders, and cancers [132]. Reversibility of protein lipidation allows multiple regulatory scenarios during protein lifetime. In most cases, the acyl chain attached to the protein is unknown; however, there is some experimental evidence showing that palmitoylation or myristoylation can modulate the trafficking of some potassium channels [133,134,135]. So far, covalently bound proteins-cholesterols are uncommon except in Hedgehog proteins, Smoothened, and Hh pathway co-receptor [132]. Protein phospholipid modification is also rare; to date, the only known example is the autophagy-related protein Atg8/LC3 [136,137]. 

Ubiquitination is a physiologically common interaction among proteins that describes the covalent attachment of ubiquitin to Lys residue in a target protein. Ubiquitination complexity is related to degradation, particularly in the ubiquitin–proteasome and autophagy–lysosome pathways. Alterations in the ubiquitin system lead to the development of many diseases [138].

### 3.3. Membrane Protein Expression and Stability

**Figure 2:**
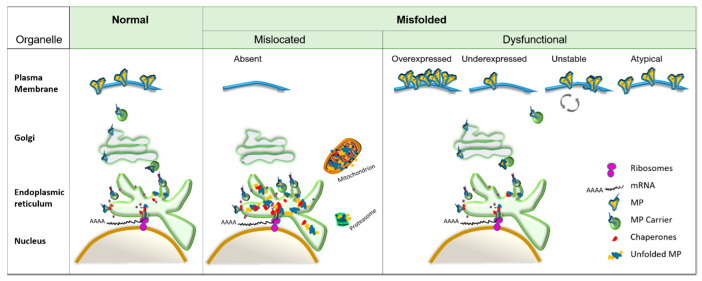
Misfolded membrane protein phenotypes. Simplified scheme of membrane proteins (MP) with folding problems. Normal: As soon as a nascent protein starts being translated at the ribosomes and translocated to the ER, chaperones will assist in folding, and well-folded proteins will travel in lipidic vesicles (MP carrier) from the ER to the Golgi apparatus to reach the plasma membrane, where they undergo a constant recycling cycle. Mislocated: Abnormally folded proteins will selectively be excluded from transport vesicles for accumulation in the lumen of the ER, triggering a heightened state of ER stress; to relax the stressed ER, the proteasomal degradation of proteins will be induced. Protein aggregates can also induce mitochondrial stress, promoting apoptosis. Dysfunctional: Function of misfolded protein reaching the PM can be affected due to protein over- or underexpression; if protein expression seems normal, protein stability in the plasma membrane may be affected, increasing the recycling turnover rate, or function can be atypical (i.e., non-functional or having a differently modulated function).

In general, protein misfolding can generate two different protein phenotypes (Figure 2): (1) Mislocalized proteins, which commonly induce cellular stress due to improper degradation [139] or structural alterations that establish novel toxic functions, sometimes inducing ER and mitochondrial stress leading to apoptosis [139,140,141] or amyloid accumulation [142,143,144,145,146,147,148,149], or (2) dysfunctional proteins due to an increased (overexpressed) or decreased (underexpressed) amount of protein reaching the PM. This phenotype occasionally disrupts protein stability at the PM (rendering proteins unstable) because of increased turnover via endocytic and recycling mechanisms; in addition, misfolded proteins that reach the PM frequently exhibit altered (atypical) functionality, such as underexpression, overexpression, or complete loss of function [150,151]. In some cases, the functionality of misfolded proteins is lost because of their mislocalization rather than the loss of the intrinsic ability of the mutant receptor to interact with its ligands or effectors.

The native forms of most proteins are in a maximum stable thermodynamic state [152]. However, many proteins are metastable, meaning that thermodynamic stability can also be achieved by using alternative folding pathways, thus inducing the formation of inactive conformations; this mechanism is crucial for regulating the biological function of proteins [152,153,154,155,156,157]. Therefore, under mild destabilizing conditions, proteins have an inherent tendency to misfold and aggregate and, hence, lose functionality. Protein levels are thus tightly regulated intracellularly and extracellularly; however, under some circumstances, such as aging, mutation, and environmental stress [158,159,160], misfolded proteins can have inappropriately exposed hydrophobic surfaces that are normally buried in the interior of the protein, leading to nonnative conformations that can interact with each other to form aggregates [161].

## 4. Physiological Consequences of Protein Misfolding

### 4.1. Effects on Membranes

The arrangement of amino acids and the exposure of hydrophobic residues are important factors affecting the ability of a protein to interact with a lipid membrane and can induce membrane fusion and destabilization. Several studies have shown that specific lipids (also called “lipochaperones”) can perform a chaperone-like function in insertion and folding, guiding the assembly of MPs [161,162]. Changes in the cell membrane affected by misfolded proteins can vary depending on the nature of the proteins and the type of lipids involved. Some proteins, particularly those characterized by electrostatic interactions, merely disrupt the membrane by the binding of positively charged amino acid residues to negative or polar lipid head groups. Such disturbances are likely reversible and short-term for some proteins [163,164,165]. In addition to electrostatic disturbance, the insertion of misfolded protein can affect membranes in several ways:

(A) Vesicle formation. The exit signals that direct proteins out of the ER for transport to the Golgi apparatus and beyond are poorly understood. Signal-dependent transport is mediated by the recognition of discrete export signals on the cargo molecule by specific receptors concentrated at specific vesicle binding sites. Thus, to be exported from the ER to the functional location, proteins must be properly folded and completely assembled, while misfolded proteins will remain in the ER bound to chaperone proteins, which may obscure the exit signals or anchor the proteins to the ER, disturbing MP trafficking into vesicles [166,167,168,169]. 

(B) Loss of membrane integrity. Different lipid compositions can alter the bilayer’s natural thickness. To shield hydrophobic surfaces from the aqueous environment, some membrane tension can be induced if the hydrophobic surface of an MP is thicker or thinner than the hydrocarbon core of the bilayer [170,171]. The arrangement of hydrophobic amino acid residues in a protein is fundamental to favor protein–lipid interactions, and misfolding may affect the shape or thickness of the membrane around the protein [172]. 

(C) Formation of pathologic ion channels. To minimize exposure of the hydrophobic regions to the aqueous medium, a hydrophobic protein may change its structural conformation to act as a bridge, allowing specific lipid interactions in the membranes and thus increasing the likelihood of ion channel formation, regardless of the native protein’s secondary structure [173]. 

### 4.2. Effect on Protein Structure and Assembly

The composition and spatial arrangement of the subunits integrating functional proteins are a prerequisite for protein function and trafficking to the cell membrane [31,171]. The assembly of diverse proteins to perform a specific function is possible depending upon cell type-specific subunit expression, as subunit assembly can be controlled in a developmentally regulated manner or in response to cellular activity. Protein assembly relies on subunit stability in the ER, intersubunit affinities, and potential subunit diffusion within the ER membrane. The homomeric structure is the simplest type of protein assembly; it can be integrated by self-assembly of repeated copies of the same subunit or formed from heteromeric complexes made of multiple distinct protein subunits [174]. Incompletely assembled complexes are usually selectively retained. The association of two or more polypeptide chains to form nonfunctional structures is defined as misassembly and is usually, by definition, a consequence of misfolding [175].

Misfolding and misassembly typically occur in the ER but can also affect other organelles, for example, (1) defective peroxisomal assembly is associated with inherited human diseases (commonly called peroxisomal biogenesis disorders), such as the severe cerebrohepatorenal Zellweger syndrome (ZS). PBDs are mainly caused by mutations in *PEX* genes codifying for peroxins, proteins responsible for normal peroxisome assembly and functions [176,177]. (2) Some transport vesicles carry cargo molecules transporting misassembled proteins to the Golgi apparatus or beyond. Proteins retained in the Golgi apparatus can also be targeted by lysosomes for degradation, suggesting that additional quality control checkpoints could act in this compartment [178,179]. (3) Misassembled proteins can be correctly targeted for degradation and transported out of the ER. When the degradation machinery fails, misassembled proteins accumulate in the cytosol as aggresomes [180]. 

Even when the loss of proteostasis underlies aging and neurodegeneration characterized by the accumulation of protein aggregates and mitochondrial dysfunction, the relationships among these negative factors are unclear. Evidence suggests that some cytosolic proteins susceptible to aggregation are imported to the mitochondria, which seems to act as a guardian of cytosolic proteostasis [181]. 

Conformational changes in the structure of a mutated protein lead to the formation of a partially folded intermediate. Intermediate states in protein folding naturally occur even in wild-type proteins. Folding/unfolding transitions thermodynamically and kinetically follow multiple discrete steps that can sometimes provide a starting point for aggregation [182]. When protein monomer aggregates produce fibrillar structures rich in β-strand conformations, the formed structures are usually called amyloid aggregates. Amyloidogenic proteins have been related to normal physiological functions such as bacterial biofilm formation or regulation of synthesis and storage of melanin in human. Amyloid aggregates are frequently related to neurodegenerative diseases that include Alzheimer’s disease, Parkinson’s disease, various tauopathies, and other human disorders [183]. Although there is no clear evidence that PM protein can form amyloid aggregates, experimental evidence shows pore formation in the bilayer due to the amyloid aggregate–lipid interaction [184,185,186], toxically affecting membrane permeabilization and ion homeostasis. In some cases, misfolded proteins and their aggregates can be re-solubilized and re-folded by protein chaperones or degraded by the ubiquitin–proteasome pathway [187]. Scientists are starting to understand the molecular mechanisms underlying the development of misfolding diseases. Mutations affecting the activity of chaperones to prevent protein misfolding, along with the production of harmful proteins and the formation of misfolded protein aggregates, may induce the toxicity associated with these pathological disorders [188,189]. 

The idea that misfolded proteins can be rescued comes true because several strategies, including chaperone overexpression or using proteasome inhibitors, facilitate the biogenesis and surface expression of these wasteful proteins, indicating that they were viable folding intermediates able to integrate into functional pools [190].

## 5. Aid for Misfolded Proteins

In 1978, Ronald Laskey and his colleagues were the first to describe a chaperone protein necessary for nucleosome assembly (nucleoplasmin) [191]. Chaperones can be expressed either in different regions or in a tissue-specific manner, and the target proteins binding to chaperones are usually termed clients [192]. Even when the protein structure is determined through the amino acid sequence, chaperones are essential regulators stabilizing protein structure and participating in the folding and assembly processes. Chaperones can bind to nascent or unfolded proteins to stabilize their structure and prevent the formation of aggregates within the cell [193,194,195]. To date, different kinds of proteins and small molecules that can be used physiologically to facilitate protein folding and assembly have been identified. Chaperones are formally classified as molecular, chemical, and pharmacological chaperones according to their origin [196,197]. 

### 5.1. Molecular Chaperones Associated with Plasma Membrane Protein Biogenesis

Molecular chaperones are a family of proteins that can recognize conflicts among paired-contact interactions in proteins, where the implicated amino acids cannot reach a minimal free energy state while folding (i.e., a frustrated structural state) [198]. Most of these chaperones were discovered as proteins expressed in response to temperature changes or environmental stresses and were named heat shock proteins (Hsp) [199]. 

Chaperones can work as holdases (holding a partial folding state of a protein), foldases (assisting protein folding in an ATP-dependent manner), or unfoldases (unfolding transiently unfolded intermediates to allow protein refolding) [200,201]. The mechanism by which chaperones recognize and assist their client proteins is still not clear as most chaperones are promiscuous molecules. Although some interactions can be regulated in an ATP-dependent manner, others cannot, suggesting that the molecular mechanism of chaperones may not be unique. 

Studies have demonstrated the interaction of chaperones with polypeptides during folding—through hydrophobic amino acid residues—to avoid aggregate formation and stabilize the structure [202,203]; however, the way chaperones interact with native folded proteins is not clear. A computational high-throughput analysis of the Hsp90 client (an ATP-dependent chaperone) failed to identify a consensus motif allowing interaction [204]. Recently, researchers solved the interaction of bacterial chaperones (e.g., ATP-independent holdases Spy, SurA, and Skp, and the ATP-dependent chaperone GroEL) with clients, demonstrating that chaperone–client interaction occurs regardless of sequence motif or local structure, but binding happens locally through an entropy-based mechanism when a protein surface is frustrated [205,206] and unable to achieve a minimum energy structural state [207,208]. Researchers have suggested several transient local interactions with some segments of the client during folding to reach a minimally frustrated conformation [198].

Hsp function is crucial because these proteins promote cell stability under stress or pathological conditions. Cells would suffer irreversible damage or even cell death without Hsps [209]. Twenty different protein families exhibit chaperone activity. These proteins are classified by their molecular weight and offer different alternatives for correct folding. To date, just a few molecular chaperones have been associated with PM biogenesis.

#### 5.1.1. Hsp70

Binding immunoglobulin protein (BiP), a member of the heat shock protein 70 (Hsp70) family, has been found in the ER lumen, where it plays a role in the recognition of misfolded MPs. Hsp70 folding is assisted by other chaperones such as Hsp90 and Hsp40, DNAJA1, and DNAJB1. Hsp70 unfolds proteins through ATP hydrolysis, inducing the cyclic binding and releasing of hydrophobic amino acids [210,211].

The balance between ER export and retention mediated through Hsp70–Hsp90 cytosolic chaperone systems [189,212] has been evident in the trafficking of mutant ion channels with impaired traffic, such as voltage-gated delayed rectifier potassium channel, hERG (human ether-a-go-go-related gene), which is related to congenital long QT syndrome type 2 (LQT2) [213], and cystic fibrosis transmembrane conductance regulator (CFTR), a chloride channel that plays an important role in the maintenance of ion balance. CFTR favors epithelial surface hydration prominently in the lung airways and pancreas, and its dysfunction is implicated in cystic fibrosis [189,214].

In vitro co-expression of Hsp70 or Hsc70 (heat shock cognate protein, a constitutively expressed member of the Hsp70 family) with mutant hERG or voltage-sensitive potassium channel Kv1.5 prolonged the channel lifetime and increased functionality at the cell surface, decreasing its ubiquitination [215]. However, overexpression of Hsp70 with the co-chaperone DNAJB1 or Hsp73 only induced modest traffic and stabilization improvement of the deletion of phenylalanine at position 508) in the CFTR channel (ΔF508-CFTR) [216,217]. Additionally, Hsc70 and one of its co-chaperones, DNAJA1, are more associated with ΔF508-CFTR than with CFTR wild-type, suggesting that chaperones engage in a mutant channel trying to refold it [218]. Mixed tetrameric channels integrated by acid-sensing ion channel subunits (ASIC1 or ASIC2) together with subunits forming an epithelial sodium channel (ENaCα or ENaCγ) are thought to form the active ASIC channels at the glioma cell surface [219,220]. Hsc70 was found to associate with ASIC2 in glioma cells [221]. Knocking down this chaperone reduced the channel activity but increased the cell surface expression and inhibited glioma cell migration [219].

Thus, these chaperones promote protein damage recovery and improve cell viability. From another functional perspective, overexpression of Hsp70 also suppresses phenotypes related to protein aggregation in models of Alzheimer’s disease [222] and Parkinson’s disease [223,224,225].

#### 5.1.2. Hsp90

The family of Hsp90 chaperones comprises critically conserved proteins that are major molecular chaperones within eukaryotic cells. Hsp90 proteins are important for cellular stabilization processes involving signal transduction, cellular trafficking, chromatin remodeling, cell growth, differentiation, and reproduction [226,227,228,229,230]. Hsp90 interacts with specific proteins through an ATP-dependent cycle, guaranteeing folding, transport, and/or assembly of the client into multiprotein complexes [231,232]. In addition to assisting in the folding of nascent proteins, Hsp90 chaperones promote protein refolding and aberrant protein degradation, possibly indicating that Hsp90 proteins can adapt their conformation to match every client or that they recognize different clients in different conformations [233,234,235,236]^.^

Hsp90 client proteins include transcription factors (e.g., HIF1α, ATF3, and p53), steroid hormone receptors (e.g., estrogen, glucocorticoid, and progesterone receptors), and kinases (e.g., EGFR, B-raf, and SRC), among other proteins. Many of these client proteins are commonly overexpressed and/or frequently mutated in cancer cells [237,238,239].

Early studies identified Hsp90 in complexes with hERG or CFTR channels. Hsp90 inhibition impaired hERG trafficking, but the effect on CFTR remains unknown [240]. 

Hsp90β overexpression restored PM expression of the mutated voltage-gated potassium channel (KCNQ4) related to autosomal dominant deafness type 2A and increased the activity of the WT channel, but not the activity of WT and mutant (W276S) mixed channels, suggesting that chaperone affinity is affected due to the mutation [241].

Since Hsp90 regulates the stability of oncoproteins important in tumor development and progression, in addition to controlling other pathological oligomeric aggregates causing neurodegenerative diseases, Hsp90 inhibitors have been suggested as a potential therapy for misfolding-related diseases [242,243]. 

Under proteotoxic stress conditions, or when cellular protein degradation is overwhelmed, misfolded MPs can become stuck and intracellularly form aggresomes [244], which should be eventually cleared through autophagy over kinetically controlled chaperone-assisted folding and degradation systems [244,245], including a group of molecular chaperones working together with other molecules with chaperone activity (e.g., calnexin and a protein disulfide isomerase that catalyzes the formation of disulfide bonds, allowing proteins to fold) [246,247,248,249].

How misassembled transmembrane domains are recognized is not clearly understood. However, because protein maturation is a complex procedure, it is expected that a recently proposed conformational frustration state recognized by chaperones [198,207,208] may consider intrinsic structural properties of the misfolded proteins [89,250]; for instance, single amino acid mutations within protein domains could potentially disrupt native helical packing interactions inducing solvation of TM segments that are naturally hydrophobic [251,252], thus prompting the formation of different protein contacts or interactions affecting their structure. 

BiP activity was experimentally evidenced for two proteins needing a β subunit to stabilize their membrane location: the Na^+^/K^+^ ATPase [251,253,254] and the single-pass TM α subunit of the αβ T-cell receptor (αβTCR) [255]. For both proteins, the lack of a β subunit left some residues of the α subunit out of the membrane, thus favoring protein interaction with BiP—targeting proteins for degradation—and reducing the probability of exporting immature proteins.

#### 5.1.3. Co-Chaperones Cooperating in Membrane Protein Folding 

Cooperation between molecular chaperones to control protein synthesis and degradation is crucial for cell maintenance. The physiological role of chaperones is evident because proteins are not always able to attain their correct conformation spontaneously, and some factors, such as age, disease, or cellular stress, may also influence protein folding, thus affecting the balance of protein ‘‘rescue’’ or protein degradation. Many co-chaperones have been reported to regulate chaperone activity related to degradation. Particularly, a highly conserved cytoplasmic protein called CHIP (carboxyl terminus of Hsc70 interacting protein) was identified during protein screening, in addition to the tetratricopeptide repeat (TPR) [256], a very conserved domain in several co-chaperones. TRP allows CHIP interaction with Hsp70, Hsc70, or Hsp90 to induce client substrate ubiquitylation and proteasome degradation because CHIP works as a ubiquitin ligase [257]. 

Some studies showed that CHIP-dependent polyubiquitination serves as a sorting signal for internalization and lysosomal degradation of the MP, including mutants from dopamine receptor D4.4, vasopressin V2 receptor (V2R), CFTR and G-protein coupled receptors (GPCRs) [258]. Later on, a supportive study showed that in vitro co-expression of CHIP with voltage-gated potassium channel Kv1.5 increased channel ubiquitination and decreased the protein level, while CHIP suppression increased channel expression [259].

Many mutations in genes encoding structural proteins may not influence protein functionality but may still cause some diseases by either arresting mutant proteins intracellularly or preventing the cellular trafficking machinery from transporting the mutant protein to an appropriate subcellular location to avoid protein misfolding and aggregate formation in the first step of the pathological cascade. Considering that chaperones are essential to many physiological processes by preventing protein misfolding and aggregation, several strategies based on their buffering capacity are rapidly emerging as promising treatments for misfolding-related diseases [196,197].

The ability to restore the biosynthesis of misfolded proteins has been demonstrated through different strategies. For instance, ΔF508-CFTR channel is a classic example of a mutation causing a misfolding disease. Nevertheless, crystal structures and biophysical studies comparing WT and mutant CFTR domains suggest only local structural changes due to the amino acidic deletion [260,261]. Several studies have demonstrated that F508 deletion induces ER retention, proteolytic degradation, and absent Cl^-^ conductance. The ability of CFTR to traffic to the PM at 37 and <30 °C was evaluated, and the lower temperature was found to favor PM localization and function [262]. Many other chaperones have been evaluated as potent modulators of several misfolding diseases. 

### 5.2. Chemical Chaperones

Some small organic molecules helping to maintain proper proteostasis in stressful environments are considered chemical chaperones because they enhance the folding and/or stability of proteins. These molecules can be present in a diverse range of organisms or tissues under denaturing conditions [263] and are potentially helpful in treating conformational diseases. Nonspecific interactions with unrelated proteins make them not specific for a therapeutic target. Some examples of these compounds are polyols, such as glycerol, dimethyl sulfoxide (DMSO), trimethylamine (trimethylamine N-oxide [TMAO]) and amino acid derivatives, 4-phenylbutyric acid (4PBA), membrane-permeable forms of promiscuous enzyme antagonists, ligands, and substrates. Thus far, the mechanism by which chemical chaperones modulate folding energy landscapes is not clearly understood [264], but these chaperones stabilize misfolded proteins, thus decreasing the formation of protein aggregates, preventing unnecessary interactions with other proteins and altering the activity of other chaperones such that proteins are transported to their final destination with increased efficiency. Chemical chaperones of the polyol TMAO and 4PBA groups act on multiple proteins, whereas antagonists, ligands, and substrates affect specific proteins (and are commonly called pharmacological chaperones) [265]. 

Chemical chaperones can be classified into two groups: osmolytes and hydrophobic compounds. Even when they lack specificity, these molecules usually have an effect only at high concentrations and are thus frequently rejected as therapeutic agents even when they rescue the misfolded protein state in vitro [190,261]. De novo design of chemical chaperones with increased activity and specificity is desirable to ameliorate protein misfolding and aggregation in different contexts.

Osmolytes: The group of osmolyte chaperones comprises low molecular weight compounds, including free amino acids and amino acid derivatives (glycine, taurine, and β-alanine). Other osmolyte compounds are polyols, such as glycerol and sucrose, and methylamines, particularly TMAO. Osmolyte chaperones are crucial for organisms exposed to stressful conditions such as fluctuating salinity, desiccation, or extreme temperatures [266]. These types of chaperones increase protein stability without disrupting protein function. Polyols protect cells against extreme conditions such as increased temperature or dehydration. Amino acids preserve proteins in high-salinity environments, and methylamines protect cells against the denaturing effect of urea [266,267]. 

Although osmolyte chaperones have effects on different kinds of proteins and under diverse conditions, they share the function of stabilizing protein structure by modifying the solvent properties. Osmolyte chaperone solvation decreases water activity around each polypeptide, forcing partially exposed hydrophobic patches to reach the most stable conformation and facilitate protein folding [268,269,270]. 

Several studies have shown that small organic compounds which stabilize PM protein [98] can rescue protein folding defects by increasing traffic and function at the PM for selective mutants on the cystic fibrosis-related CFTR chloride channel [98,271], aquaporin-2 water channel (AQP2), and V2R associated with nephrogenic diabetes insipidus (NDI) [272,273]. Some of the mutants in these studies were also tested with either TMAO, DMSO, or glycerol, showing different rescuing effects. TMAO showed a lower ability to rescue MPs, which may be due to an enhanced hydration potential that affects the reagent permeability on lipidic membranes or the stability of hydrophobic regions on the MP [274,275].

Hydrophobic compounds: Several molecules have been classified as hydrophobic chaperones, including (4PBA) and bile acids (e.g., oxysterol, an oxygenated isoform of cholesterol).

4PBA is capable of restoring the cell surface expression of mislocated mutants on bile salt export pump (BSEP), which is related to an inherited autosomal recessive liver disease called progressive familial intrahepatic cholestasis type 2 (PFIC2) that leads to cirrhosis and death before adulthood [276]. In a recent study testing a multidrug regimen of 4PBA mixed with anticonvulsants oxcarbazepine and maralixibat, PFIC2 symptoms were controlled in two siblings with partial loss of BSEP activity [277]. 4PBA has also been tested with the mutant channel Δ508-CFTR-inducing cell surface expression [278], and some clinical trials using this chemical chaperone in cystic fibrosis patients have demonstrated an improvement in CFTR function in the nasal epithelia [279]. 

For cyclic nucleotide-gated channels related to retinopathies, reduced degradation and/or promoted PM localization of defective subunits was shown using chemical chaperones such as 4PBA or the bile acid component tauroursodeoxycholic acid (TUDCA) [280].

The general mechanism of action proposed for hydrophobic chaperones is based on the interaction between the chaperone’s hydrophobic regions and the exposed hydrophobic segments of the unfolded protein. However, even though 4PBA and bile acids can reduce aggregate accumulation in vivo and in vitro and revert ER stress, these molecules may be more complex in the action mechanism influencing different levels of regulation [196].

### 5.3. Pharmacological Chaperones

Pharmacological chaperones, also known as pharmacoperones, are lipophilic compounds stabilizing protein conformation to prevent degradation and promote proper trafficking to their functional site of action in the cell. These molecules represent one of the most promising therapeutic strategies to treat misfolding-related diseases [281]. Unlike chemical chaperones, these low molecular weight compounds bind selectively to proteins, stabilizing the protein structure and restoring protein localization and function. Enzymes, agonists, antagonists, and some synthetic compounds are examples of pharmacoperones [282]. 

Antagonists used as chaperones should ideally provide an effective rescue response at the lowest concentration, without losing the ability to dissociate from the rescued protein to facilitate the endogenous ligand binding [283]. Antagonists are highly efficacious in rescuing mislocated mutant proteins by preventing agonist or substrate access [284,285,286]; however, antagonists of high affinity for receptors and ion channels may yield nonfunctional channels expressed at the PM, as was shown for the rescued ATP-sensitive potassium channels (KATP) treated with sulfonylureas [287].

Agonist molecules were identified as pharmacoperones when SR49059, a small, cell-permeable molecule formerly developed as a vasopressin antagonist, was able to rescue the function of ER-retained V2 vasopressin receptor (V2R) mutants [288,289]. The experiments clearly showed significantly improved kidney function in NDI patients [289]. Rhodopsin-like G-protein-coupled MC4R (melanocortin 4 receptor), which causes severe early-onset morbid obesity in humans, exemplifies misfolded and intracellularly retained proteins rescued using antagonists [290].

Channel blockers also represent an alternative to modulate protein folding and trafficking; cisapride, E-4031, and the non-specific antiarrhythmic drug quinidine have experimentally rescued mutant hERG leading to LQT2. However, using channel blocker as a pharmacological strategy is still controversial as blocking ion flow can affect K^+^ flux and cell homeostasis, leading to a prolonged QT interval and increased risk of developing arrhythmia [291,292,293].

The dissociation rate is not essential for using an agonist as a chaperone [294,295]. Mislocation of mutant MPs has been rescued using agonists. Defective trafficking of misfolded mutant CNG channels has been successfully rescued using a cell-permeable cyclic nucleotide agonist [280]. High-affinity non-peptide agonists were able to selectively rescue PM expression and function of misfolded arginine-vasopressin receptor 2 (AVPR2) mutants associated with NDI [296]. Some mutants in the calcium-sensing receptor (CaSR) leading to familial hypocalciuric hypercalcemia and neonatal hyperparathyroidism can also be rescued using membrane-permeant allosteric agonists to recover protein functionality [190,295].

In addition to functioning as cell proteostasis modulators modifying the cell proteome and increasing MP maturation, pharmacological chaperones can also act as correctors and potentiators. In fact, for the CFTR mutant channel, it has been tested that some compounds, such as corr-4a and VRT-532, interact directly with the misfolded protein to correct the biosynthetic pathway and enhance trafficking and channel function [297]. VX-809 (Lumacaftor) is a corrector tested by itself or in combination with other molecules [298] in patients with cystic fibrosis and is a potential treatment for other pathologies associated with misfolding and misrouted proteins, as has been proved to Stargardt disease, which invariably ends in legal blindness (visual acuity of 20/200 or less [299]) [298]. Among other correctors or pharmacoperones, Lumacaftor represents hope to set up therapeutic strategies for misfolding diseases [300,301]. 

Assistance to stabilize protein folding can be analyzed in vivo or in vitro, as proteins can naturally interact with many compounds intracellularly. Proteins can be assisted by chemical chaperones, which helps scientists to better understand how proteins are properly folded in vitro and thus find more suitable and specific compounds (or pharmacological chaperones) to develop a therapeutic strategy for misfolding diseases.

Table 2 summarizes the misfolded PM proteins related to pathologies and the therapeutic approaches used; molecular, chemical, and pharmacological chaperones are widely regarded as a promising therapeutic strategy for these pathologies.

## 6. Conclusions

MP localization is critical for normal cell physiology. The correct routing of proteins is as important as the genetic machinery for protein expression. Protein trafficking, moreover, is not a simple or unidirectional process. Naturally, trafficking assistance from the ER to the plasma membrane involves many proteins and factors as protein carriers or chaperones of molecular cargo, and, as a response to misfolding, chaperones may also mask harmful modifications in the folding energy landscape. However, when protein folding proceeds incorrectly, many diseases can arise; a wide range of diseases result when protein misfolding induces intracellular retention of MPs. Misassembled proteins that reach the membrane can also lead to disease due to dysfunctionality.

Currently, specific therapies for conformational diseases are lacking because of a gap in the understanding of the mechanisms by which the natural conformation of proteins is altered into many misfolded pathological forms. However, encouragingly, an extensive and concerted effort is already underway to combat misfolding diseases. The discovery of compounds with therapeutic chaperone ability is customarily initiated through high-throughput screening of libraries, including natural or synthesized compounds, searching for stabilizing binders using in silico, in vitro, in vivo, or cell-based approaches. As we continue increasing knowledge of disease mechanisms, we also continue to discover molecules that could interact with PM proteins, mimicking the effect of natural chaperones that correct misfolding.

## Figures and Tables

**Table 1:** Signal peptide for protein localization in the endoplasmic reticulum and plasma membrane.

Sequence	Protein	Location	Organism	Reference
VVQAITFIFKSLGLKCVQFLPQVMPTFLNVIRVCDGAIRE.	mTOR.	Endoplasmic reticulum.	Homo sapiens; Mus musculus; Rattus norvegicus.	[39]
HALSYWKPFLVNMCVATVLTAGAYLCYRFLFNSNT.	PTP-1B.	Endoplasmic reticulum.	Homo sapiens.	[40]
MEAMWLLCVALAVLAWG.	GlcNAc-PI.	Endoplasmic reticulum.	Homo sapiens.	[41]
IPHDLCHNGEKSKKPSKIKSLFKKKSK.	STIM2.	Endoplasmic reticulum.	Homo sapiens; Mus musculus.	[42]
GVMLGSIFCALITMLGHI.	Cosmc.	Endoplasmic reticulum.	Bos taurus; Homo sapiens; Mus musculus; Rattus norvegicus.	[43]
MRLLLALLGVLLSVPGPPVLS.	FGFR4.	Plasma membrane.	Homo sapiens.	[44]
MDCRKMARFSYSVIWIMAISKVFELGLVAG.	TDGF.	Plasma membrane.	Homo sapiens.	[45]
MPAWGALFLLWATAEA.	(GP)IX.	Plasma membrane.	Homo sapiens.	[46]
LRCLACSCFRTPVWPR.	prRDH.	Plasma membrane.	Bos taurus.	[47]
MGCGCSSHPE.	Lck.	Plasma membrane.	Homo sapiens; Aotus nancymaae.	[48,49]
VTNGSTYILVPLSH.	FSHR.	Plasma membrane.	Homo sapiens.	[50]
AETENFV.	M3 mAChR.	Plasma membrane.	Homo sapiens; Gorilla gorilla gorilla; Pan troglodytes; Pongo pygmaeus.	[51,52]

**Table 2:** Chaperones used to rescue misfolded plasma membrane proteins related to diseases.

Misfolded Membrane Protein	Disease	Gene	Rescuing Strategy		
			Molecular Chaperones	Chemical Chaperones	Pharmacological Chaperones
α-synuclein	Parkinson’s disease	SNCA	Hsp70 [223,224,225]		
Aquaporin-2	Autosomal Nephrogenic Diabetes Insipidus	AQP2		Glycerol, Trimethylamine-N-oxide (TMAO) and Dimethyl sulfoxide (DMSO) [2,272,273].	
Arginine-Vasopressin (AVP) Receptor 2 (AVPR_2_)	Nephrogenic Syndrome of Inappropriate Antidiuresis and Diabetes Insipidus (nephrogenic, X-Linked)	AVPR2			OPC51803, VA999088, and VA999089 [293]. L44P, A294P, and R337X [294]. SR49059, VPA-985, OPC31260, OPC41061 (Tolvaptan) and SR121463B [293,302,303]. MCF14, MCF18, and MCF57 [294,304].
ATP-binding Cassette Transporter	Tangier disease	ABCA1		Sodium 4-Phenylbutyrate (4-PBA) [305].	
	Stargardt Eye disease	ABCA4			VX-809 (Lumacaftor) [306].
Bile Salt Export Pump (BSEP)	Progressive Familial Intrahepatic Cholestasis type 2	ABCB11		4-PBA mixed with Anticonvulsant-Oxcarbazepine, and Maralixibat [274,275].	
Calcium-Sensing Receptor (CaSR)	Familial Hypocalciuric Hypercalcemia	CaSR			MG132, NPS R-568 [295,307].
Cardiac Sodium (Na^+^) Channel NaV1.5	Brugada Syndrome Nocturnal Death syndrome	SCN5A		Curcumin [308].	Mexiletine [309].
Connexin Cx31, Cx43, Cx50	Charcot-Marie-Tooth syndrome	GJA1			Cycloheximide [310].
Copper-transporting P-type ATPase	Menkes disease	ATP7A		Excess of copper [311].	Copper Toxicosis Protein COMMD1 [311].
Cyclic Nucleotide Gated (CNG) Channel	Retinitis Pigmentosa, Achromatopsia	CNGA3		TUDCA (Tauroursodeoxycholate Sodium salt), 4-PBA [278]. Glycerol [312]. MTSHB (Hydroxybenzyl-Methanethiosulfonate), MTSEA (Aminoethyl-Methanethiosulfonate) [123].	CPT-cGMP [8-(chlorophenylthio)-cGMP] [278].
Cystic Fibrosis Transmembrane Conductance Regulator (CFTR)	Cystic Fibrosis	CFTR	Hsc70, Hsp90 [313]. Hsc70/Hdj-2 [218].	Glycerol [98]. TMAO [269]. 4-PBA [276,277].	VX-809 (Lumacaftor) [269]. Lumacaftor/Ivacaftor [314,315]. Cycloheximide [316]. Corr-4a and VRT-532 [317,318]. VX-661(Tezacaftor)/Ivacaftor [319].
Dopamine Transporter (DAT)	Infantile parkinsonism-dystonia	SLC6A3			Ibogaine, Noribogaine [320,321,322].
Gonadotropin Releasing Hormone Receptor (GnRHR)	Hypogonadotropic hypogonadism	GNRHR	JB12, Hsp70 [323].		IN3 [324].
HERG potassium channel	Hereditary long QT syndrome	KCNH2	sp40/DnaJ [325].		E-4031 [289,326]. Cisapride [291]. Thapsigargin [290].
Insulin receptor	Diabetes Mellitus, Insulin-resistant syndrome	INSR	Calnexin and Calreticulin [327].		
Melanocortin-4 receptor (MC4R)	Severe early-onset morbid obesity	MC4R			Ipsen 17 [288,328]. ML00253764, Ipsen 5i [328,329,330].
Voltage-gated potassium channel (VGKC)	Autosomal Dominant Deafness type 2A	KCNQ4	Hsp90β [241].		
Neuroligin-3	X-linked autism, Asperger syndrome	NLGN3	Calnexin [331].		
Pendrin	Pendred syndrome and Non-syndromic Hearing loss	SLC26A4 (PDS)		TMAO [332].	Cycloheximide (CHX), Puromycin [332].
Prion Protein (PrP)	Genetic Creutzfeldt-Jakob disease, Gerstmann Straussler Scheinker syndome and Fatal Familial Insomnia	PRNP	BiP [333], [334].		
Rhodopsin	Retinitis Pigmentosa	RHO	1-*cis* retinal [335].	DMSO [336]. Curcumin [337].	YC-001 [338]. S-RS1 [336].
Sodium-borate cotransporter	Corneal dystrophy	SLC4A11			Anti-inflammatory drugs (NSAIDs), Glafenine, Ibuprofen, and Acetylsalicylic acid dissolved in DMSO [339].
Vasopressin Type 2 Receptor (V2R)	Nephrogenic Diabetes Insipidus	V2R		Glycerol, DMSO and TMAO [272,273].	SR49059 [197,286,287]. Thapsigargin/Curcumin and Ionomycin [340].
Voltage sensitive potassium channel (Kv1.5)	Atrial Fibrillation	KCNA5	Hsp70 [215].		
